# Catastrophic injuries and exertional medical events in lacrosse among youth, high school and collegiate athletes: longitudinal surveillance over four decades (1982–2020)

**DOI:** 10.1080/07853890.2024.2311223

**Published:** 2024-02-09

**Authors:** Garrett A. Moseley, Andrew E. Lincoln, Jonathan A. Drezner, Randi DeLong, Erin Shore, Nina Walker, Johna K. Register-Mihalik, Robert C. Cantu, Kristen L. Kucera

**Affiliations:** a Department of Exercise and Sport Science, National Center for Catastrophic Sports Injury Research, University of North Carolina at Chapel Hill, Chapel Hill, NC, USA; b Special Olympics International, Washington, DC, USA; c Department of Family Medicine, Center for Sports Cardiology, University of Washington, Seattle, WA, USA; d Department of Epidemiology, University of North Carolina at Chapel Hill, Chapel Hill, NC, USA; e Department of Exercise and Sport Science, University of North Carolina at Chapel Hill, Chapel Hill, NC, USA; f Injury Prevention Research Center, University of North Carolina at Chapel Hill, Chapel Hill, NC, USA; g Matthew Gfeller Center, University of North Carolina at Chapel Hill, Chapel Hill, NC, USA; h Cantu Concussion Center, Emerson Hospital, Concord, MA, USA

**Keywords:** Epidemiology, athletic training, injury, death, sports, sudden cardiac arrest, commotio cordis, spine injury

## Abstract

**Objective:**

To determine the incidence rates (IRs) of catastrophic injuries and exertional medical events in lacrosse athletes.

**Methods:**

Catastrophic injuries and exertional medical events in lacrosse in the US among youth or amateur, high school and college athletes were analysed from the National Center for Catastrophic Sport Injury Research (NCCSIR) database from 1982/83 to 2019/20. Frequencies, IRs per 100,000 athlete-seasons (AS) with 95% confidence intervals (CIs), and incidence rate ratios (IRRs) with 95% CIs were calculated. Participation data were gathered from the National Federation of State High School Associations (NFHS), National Collegiate Athletic Association (NCAA) and USA Lacrosse.

**Results:**

Sixty-nine catastrophic events (16 youth or amateur, 36 high school and 17 college; 84% male) occurred in US lacrosse from 7/1/1982 to 6/30/2020. Thirty-six percent of all incidents were fatal. The overall IR was 0.5 per 100,000 AS (95% CI: 0.4–0.7). There were 15 cases of non-traumatic sudden cardiac arrests (SCAs) and 15 incidents of commotio cordis. Fatality rates from SCA and commotio cordis decreased 95% (IRR = 0.05; 95% CI: 0, 0.2) from 1982/83–2006/07 to 2007/08–2019/20. Incidence rates were higher for collegiate versus high school 1982/83–2019/20 (IRR = 3.2; 95% CI: 1.8, 5.7) and collegiate versus youth 2005/06–2019/20 (IRR = 8.0; 95% CI: 3.0, 21.4) level. Contact with a stick or ball (41%) and contact with another player (20%) were the primary mechanisms of injury.

**Conclusions:**

The incidence of catastrophic events during lacrosse was higher among collegiate than high school or youth athletes. SCA from an underlying cardiac condition or from commotio cordis was the most common catastrophic event. Fatality rates from catastrophic injuries have declined significantly over the study period, perhaps driven by protective measures adopted by lacrosse governing bodies.

## Abstract (plain language summary)

Collegiate athletes had a higher incidence rate of catastrophic events during lacrosse, while high school athletes had the greatest overall number of events.

Cardiac-related events were the most common catastrophic event.

Fatality rates for non-traumatic sudden cardiac arrest and commotio cordis have decreased 95% over the past several decades, perhaps related to protective measures and increased access to automated external defibrillators promoted by lacrosse governing bodies.

## Introduction

As an organized sport, lacrosse has seen a dramatic 227% increase in participation within the United States at all levels of play since 2001 [1]. In 2018, USA Lacrosse reported that there were 43,228 participants at the college level and 332,217 at the high school level, with males representing a slight majority of all participants [2]. Previous studies have acknowledged this rapid increase in participation and corresponding need to identify the injury burden in the sport in order to ensure a safe sporting environment [3–15]. These studies have primarily focused on injuries in lacrosse that are non-catastrophic in nature, such as concussions, lower extremity ligament tears and bone fractures [3–10,12–15]. Several of these studies have identified general injury trends in the sport, including a higher injury rate for males, a higher injury rate during competition as opposed to practice, and a preponderance of injuries sustained via contact with either another player or a stick/ball [2–4,6,8,9,15]. Studies focusing on catastrophic incidents in lacrosse are sparse and limited to specific catastrophic incidents such as commotio cordis (a sudden cardiac arrest (SCA) event and ventricular arrhythmia precipitated by non-penetrating blunt trauma to the chest) and sudden death. One study found lacrosse to have similar sudden death rates when compared to other major sports and identified males to be the most common victims of these events [10].

Despite the growing popularity of the sport, a comprehensive investigation of all catastrophic injuries and exertional medical events in the sport has not been conducted. As such, the objective of our study was to examine the incidence and causes of catastrophic lacrosse injuries and exertional medical events across the youth or amateur, high school, and collegiate levels to inform and improve the safety of athletic participation.

## Methods

Surveillance data from the National Center for Catastrophic Sport Injury Research (NCCSIR) was utilized to examine catastrophic lacrosse events in the US from 1 July 1982 to 30 June 2020. Since 1982, the NCCSIR has conducted surveillance of catastrophic sport-related injuries in the US [16]. Using a combination of information sources, including publicly available news reports, reports from state high school associations and schools, and reports from individuals, the NCCSIR has collected catastrophic events across youth, middle school, club, high school, collegiate, semi-professional and professional sport levels. In addition, to ensure inclusion of all eligible events and to supplement information for the events captured, the authors of this paper conducted ad hoc searches in Google, Newsbank and LexisNexis from 2 December 2020 to 4 December 2020 and from 16 June 2021 to 17 June 2021 of catastrophic lacrosse injuries that occurred during the study period. Within Google and Newsbank, the following search terms were employed: (1) ‘Lacrosse player collapse’, (2) ‘[athlete name] lacrosse death’, (3) ‘Lacrosse player death’, (4) ‘Lacrosse player heat stroke’, (5) ‘Lacrosse player exertional sickling’ and (6) ‘Lacrosse player cardiac arrest’. Within Newsbank, the authors used the following search string: lacrosse AND (college or ‘high school’ or organized or recreational or ‘middle school’ or youth) AND (death or collapse or commotio or heat or cervical or neck). Within LexisNexis, the authors used the following search string: lacrosse AND (collapse or death or commotio). All available media and autopsy reports for each incident were reviewed by the authors to ensure accurate variable coding.

The NCCSIR defines a catastrophic sport injury as any injury or medical condition that resulted in fatality, permanent disability, serious injury (fractured neck or serious head injury such as a subdural hematoma), temporary or transient paralysis, heat stroke due to exercise, exertional sickling, lightning-related injuries, SCA or severe cardiac disruption [17]. Medical conditions such as rhabdomyolysis, asthma attack and diabetic emergency were also included if the athlete was admitted to an inpatient hospital facility for treatment beyond care provided in an emergency department. To be included within this study, the catastrophic injury or medical condition had to occur at the youth or otherwise amateur, high school or collegiate level of play as a result of a scheduled team activity. Exertional and cardiac events where the athlete was engaging in personal fitness and/or conditioning directly related to participation in a sponsored sport were also included. However, it is important to note that the NCCSIR did not begin actively capturing cardiac or exertional incidents that occurred outside of organized sporting activities prior to the 2013–2014 academic year (AY2013/14), at which point the centre’s case capture strategies and case definitions were revised to include SCA incidents occurring at rest or during sleep. For the purposes of this study, these SCA incidents that occurred at rest or during sleep were not included.

The National Federation of State High School Associations (NFHS), National Collegiate Athletic Association (NCAA) and USA Lacrosse collect participation data on an annual basis. USA Lacrosse data collection for high school, collegiate and youth levels began in 2001 and extended through 2018 at the time of data analysis [18]. USA Lacrosse participation data stratified by sex for all levels of play were available starting in 2006. Annual NFHS and NCAA participation numbers were used from AY1982/83 to 2004/05 to account for all high school and college lacrosse participants [19,20]. USA Lacrosse participation data were used for all high school and college participants from AY2005/06 to 2019/20.

In this study, youth play was defined to be any event that occurred at the middle school or organized non-school sponsored (club) levels of play in athletes 17 and under, while amateur play was defined to be any event that occurred at a recreational (school club, intramural and summer camp) level of play. Accurate participation data for athletes that participated in middle school or organized non-school sponsored levels of play are limited. USA Lacrosse has provided the most comprehensive participation data for youth stratified by sex since 2006. Notably, USA Lacrosse has varied its definition of youth play over the years (15 and under prior to 2015; 14 and under since 2015). Youth rates for males and females were calculated using USA Lacrosse’s participation data starting in AY2005/06. No incidence rates (IRs) for amateur play were calculated.

The most up-to-date participation data from USA Lacrosse at the time of data analysis extended through the fall of 2018. As such, the authors used the 2018 USA Lacrosse participation numbers for both AY2018/19 and AY2019/20 in addition to AY2017/18.

Data were analysed using SAS Base 9.4 (version 14.3; SAS Institute Inc., Cary, NC) to assess IRs and patterns of catastrophic injuries in lacrosse in the US. Descriptive statistics of catastrophic injuries consisted of frequencies, survival proportions, IRs per 100,000 athlete-seasons (AS), and the associated 95% confidence intervals (CIs) for survival proportions and IRs. The University of North Carolina at Chapel Hill provides a copyright license for SAS Base 9.4 (version 14.3, Cary, NC) for all faculty, staff and students, giving the first author and several other authors access to this tool. Incidence rate ratios (IRRs) and survival incidence proportion ratios (RRs) were also calculated to compare incidence across several parameters, including sport level, sex, event type and time period. IRRs comparing youth to other sport levels were only calculated from AY2005/06 to 2019/20 due to the lack of participation data available for youth prior to AY2005/06. A simple linear regression was used to model annual IRs across the study period for college and high school from AY1982/83 to 2019/20 and for youth and all levels combined from AY2005/06 to 2019/20.

Lastly, high profile, on-field deaths of lacrosse athletes from 2000 to 2004 led to a concerted effort to improve safety and access to AEDs. USA Lacrosse promoted a cardiac safety program to increase the availability of AEDs in AY2007/08 and offered preferred pricing for AEDs [12,21–25]. The potential impact of USA Lacrosse’s cardiac safety program initiated in AY2007/08 was assessed by comparing IRs and survivor proportions before (AY1982/83 to 2006/07) and after (AY2007/08 to 2019/20). Recognizing the multivariate nature of factors affecting improvements in cardiac safety (high profile deaths, AED training programs and partnership programs), lagged analyses were conducted to assess variance in the timing of the effect of these factors (cutoff points set at: AY2005/06, AY2006/07, AY2007/08 and AY2008/09). Fatality rates before and after each cutoff point were compared with IRRs. Of note, two cardiac cases occurred among amateur athletes and were thus excluded from this analysis (one incident of non-traumatic SCA and one incident of commotio cordis). All ratio measures were considered statistically significant if the 95% CI did not cross the null value of 1.0.

## Results

From 1 July 1982 to 30 June 2020, a total of 58 catastrophic lacrosse incidents were captured by NCCSIR at the youth or amateur, high school and collegiate levels of play. Eleven additional catastrophic lacrosse incidents that occurred in the same timeframe were captured using ad hoc searches for a total of 69 incidents (16 youth or amateur (9 and 7, respectively), 36 high school and 17 college), 25 (36%) of which were fatal (Table 1).

**Table 1.** Demographic and descriptive characteristics of lacrosse athletes who suffered a catastrophic incident in the US from 1 July 1982 through 30 June 2020 (*n* = 69).

Variables	Fatality, *n* (%)	Non-fatality, *n* (%)	Youth or amateur, *n* (%)	High school, *n* (%)	College, *n* (%)	Total, *n* (%)
Total	25 (36)	44 (64)	16 (23)	36 (52)	17 (25)	69 (100)
Sex						
Female	1 (4)	10 (23)	1 (6)	3 (8)	7 (41)	11 (16)
Male	24 (96)	34 (77)	15 (94)	33 (92)	10 (59)	58 (84)
Age						
12–14 years old	5 (20)	3 (7)	5 (31)	3 (8)	0	8 (12)
15–17 years old	10 (40)	14 (32)	4 (25)	20 (56)	0	24 (35)
18–20 years old	7 (28)	8 (18)	1 (6)	9 (25)	5 (29)	15 (22)
21+ years old	3 (12)	4 (9)	2 (13)	0	5 (29)	7 (10)
Unknown	0	15 (34)	4 (25)	4 (11)	7 (41)	15 (22)
Sport level						
College	6 (24)	11 (25)				17 (25)
High school	12 (48)	24 (55)				36 (52)
Youth or amateur	7 (28)	9 (21)				16 (23)
Position						
Attack	2 (8)	6 (14)	1 (6)	5 (14)	2 (12)	8 (12)
Midfield	4 (16)	9 (21)	4 (25)	6 (17)	3 (18)	13 (19)
Defense	3 (12)	8 (18)	3 (19)	7 (19)	1 (6)	11 (16)
Goalkeeper	2 (8)	4 (9)	0	4 (11)	2 (12)	6 (9)
Unknown	14 (56)	17 (39)	8 (50)	14 (39)	9 (53)	31 (45)
Nature of injury						
Traumatic	13 (52)	36 (82)	12 (75)	27 (75)	10 (59)	49 (71)
Medical condition or exertional	12 (48)	8 (18)	4 (25)	9 (25)	7 (41)	20 (29)
Injury/illness type						
Commotio cordis	7 (28)	8 (18)	5 (31)	7 (19)	3 (18)	15 (22)
Head injury	3 (12)	6 (14)	2 (13)	7 (19)	0	9 (13)
Other traumatic injury	2 (8)	9 (21)	3 (19)	2 (6)	6 (35)	11 (16)
Rhabdomyolysis	0	5 (11)	0	0	5 (29)	5 (7)
Spinal cord injury	1 (4)	13 (30)	2 (13)	11 (31)	1 (6)	14 (20)
Sudden cardiac arrest	12 (48)	3 (7)	4 (25)	9 (25)	2 (12)	15 (22)
Player action						
Blocking shot^a^	2 (8)	11 (25)	6 (38)	5 (14)	2 (12)	13 (19)
Checking	1 (4)	6 (14)	2 (13)	4 (11)	1 (6)	7 (10)
Conditioning	2 (8)	2 (5)	1 (6)	2 (6)	1 (6)	4 (6)
Defending	0	2 (5)	0	2 (6)	0	2 (3)
General play	11 (44)	8 (18)	5 (31)	7 (19)	7 (41)	19 (28)
Goaltending	1 (4)	2 (5)	0	3 (8)	0	3 (4)
Loose ball	0	4 (9)	1 (6)	3 (8)	0	4 (6)
Other	7 (28)	0	1 (6)	6 (17)	0	7 (10)
Shooting	0	1 (2)	0	1 (3)	0	1 (2)
Unknown	1 (4)	3 (7)	0	3 (8)	1 (6)	4 (6)
Weights	0	5 (11)	0	0	5 (29)	5 (7)
Mechanism of injury						
Infection or illness	12 (48)	3 (7)	4 (25)	9 (25)	2 (12)	15 (22)
Player to player contact	2 (8)	12 (27)	3 (19)	8 (22)	3 (18)	14 (20)
Contact with apparatus or object^b^	8 (32)	20 (46)	8 (50)	14 (40)	6 (35)	28 (41)
Ball	7 (88)	16 (80)	7 (88)	10 (71)	6 (100)	23 (82)
Stick	1 (13)	3 (15)	1 (13)	3 (21)	0	4 (14)
Unknown	0	1 (5)	0	1 (7)	0	1 (4)
Contact with ground	0	2 (5)	1 (6)	1 (3)	0	2 (3)
Environmental	0	5 (11)	0	0	5 (29)	5 (7)
No direct or indirect contact	2 (8)	0	0	2 (6)	0	2 (3)
Unknown	1 (4)	2 (5)	0	2 (6)	1 (6)	3 (4)
Time of year						
January–March	5 (20)	16 (36)	3 (19)	9 (25)	9 (53)	21 (30)
April–June	11 (44)	23 (52)	5 (31)	24 (67)	5 (29)	34 (49)
July–September	5 (20)	1 (2)	4 (25)	0	2 (12)	6 (9)
October–December	4 (16)	4 (9)	4 (25)	3 (8)	1 (6)	8 (12)
Type of event						
Game	11 (44)	30 (68)	10 (63)	26 (72)	5 (29)	41 (59)
Practice	8 (32)	7 (16)	1 (6)	8 (22)	6 (35)	15 (22)
Scrimmage	2 (8)	0	2 (13)	0	0	2 (3)
Conditioning session	1 (4)	0	0	1 (3)	0	1 (2)
Strength/weight session	0	5 (11)	0	0	5 (29)	5 (7)
Other^c^	3 (12)	2 (5)	3 (19)	1 (3)	1 (6)	5 (7)

^a^ Blocking shot is a player action that is unique to field players in this paper. Player actions where goalies were blocking shots were classified as ‘Goaltending’.

^b^ Variables ‘Ball,’ ‘Stick’ and ‘Unknown’ are sub-variables under the ‘Contact with Apparatus or Object’ variable. Percent values given for these three sub-variables represent the proportion they comprise of the ‘Contact with Apparatus or Object’ variable.

^c^ The incidents that fell into the category of ‘other’ included summer camps, pre-game warmups and tryouts.

### Overall injury rates

**Figure 1.**
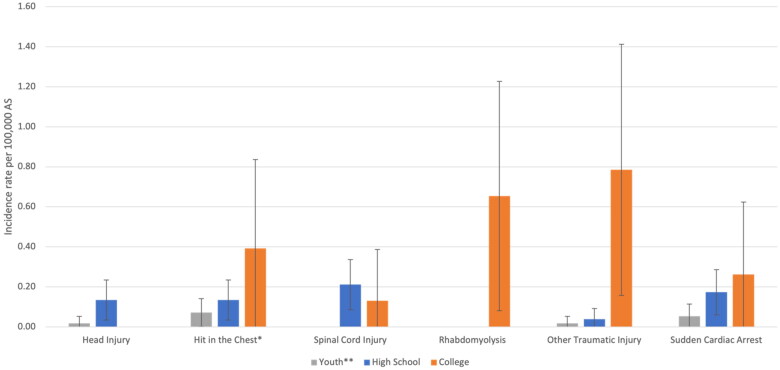
Incidence rates of catastrophic injuries and events stratified by sport level in US lacrosse: college and high school from AY1982/83 to 2019/20 and youth level from AY2005/06 to 2019/20. Vertical interval bands represent 95% confidence intervals for the incidence rate per 100,000 AS. *Hit in chest injuries include both commotio cordis and cardiac contusions. **Youth incidence rates calculated from AY2005/06 to 2019/20.

**Figure 2.**
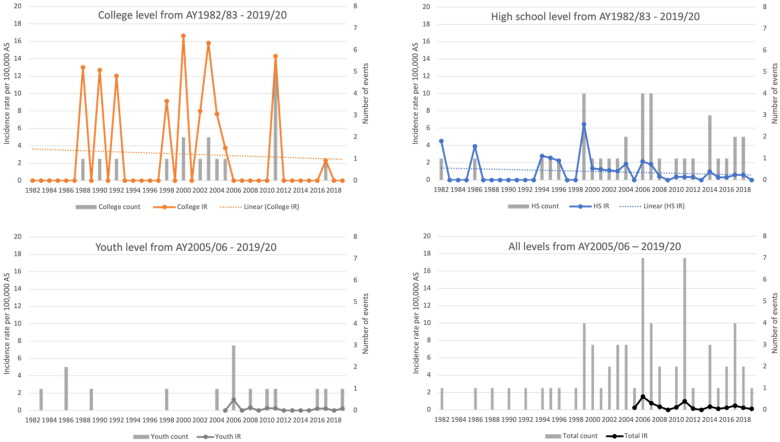
Frequency and incidence rate of all catastrophic events by academic year stratified by sport level: college and high school from AY1982/83 to 2019/20 and youth level and all levels combined from AY2005/06 to 2019/20. Simple linear regression model (dotted line is the line of best fit for the rate). (a) College AY1982/83–2019/20: decrease in the incidence rate is not significant (*p* = .6953); beta coefficient = –0.03; SE = 0.08. (b) High school AY1982/83–2019/20: decrease in the incidence rate is not significant (*p* = .3185); beta coefficient = –0.02; SE = 0.02. (c) Youth AY2005/06–2019/20: decrease in the incidence rate is not significant (*p* = .2783); beta coefficient = –0.02; SE = 0.02. (d) All levels AY2005/06-2019/20: decrease in the incidence rate is not significant (*p* = .0673); beta coefficient = –0.06; SE = 0.03.

The overall IR of catastrophic events per 100,000 AS across the study period for all levels was 0.54 (95% CI: 0.4, 0.7). The overall fatality rate was 0.20 (95% CI: 0.1, 0.3). The IR for all catastrophic events at the college level was 2.2 (95% CI: 1.2, 3.3); the IR at the high school level was 0.69 (95% CI: 0.5, 0.9); and the IR at the youth level from AY2005/06 to 2019/20 was 0.16 (95% CI: 0.1, 0.3) (Table 2). Catastrophic IRs overall were higher among college athletes compared to high school from AY1982/83 to 2019/20 (IRR = 3.2; 95% CI: 1.8, 5.7) and college athletes compared to youth from AY2005/06 to 2019/20 (IRR = 8.0; 95% CI: 3.0, 21.4) (Table 2). Fatal, non-fatal, traumatic and non-traumatic injury events displayed the same pattern that was observed for all catastrophic events: the more competitive the sport level, the higher the catastrophic incident rate (Table 2, Figure 1). This pattern held when stratified by games and practices. At the high school level, games were associated with higher catastrophic IRs compared to practices (IRR = 3.3; 95% CI: 1.5, 7.2), but this difference was not statistically significant at the college or youth levels. When considering all levels from AY2005/06 to 2019/20, the average annual IR declined 0.06 per 100,000 AS (*p* = .07, Figure 2). Similarly, average annual IRs declined at all sport levels. None of these trends were statistically significant (Figure 2).

**Table 2.** Incident rates and associated 95% confidence intervals for catastrophic events in each sport level stratified by sex, event type and overall injury category type for all catastrophic events, fatal events and non-fatal events that occurred in lacrosse in the US from 1 July 1982 through 30 June 2020 (*n* = 62).

	College rate (*n* = 17)	High school rate (*n* = 36)	Youth rate^a^ (*n* = 9)	IRR (95% CI) college vs. HS	IRR (95% CI) college vs. youth^a^	IRR (95% CI) HS vs. youth^a^
Male rate (95% CI)	2.2 (0.8, 3.6)	1.1 (0.7, 1.5)	0.3 (0.1, 0.4)	2.0 (1.0, 4.1)	2.4 (0.5, 11.2)	3.1 (1.4, 6.9)
Female rate (95% CI)	2.3 (0.6, 4.0)	0.1 (0.0, 0.3)	0	16.4 (4.2, 63.3)	–	–
IRR (95% CI) male vs. female	1.0 (0.4, 2.5)	7.8 (2.4, 25.5)	–			
Game rate (95% CI)	0.7 (0.1, 1.2)	0.5 (0.3, 0.7)	0.1 (0.0, 0.2)	1.3 (0.5, 3.4)	2.0 (0.2, 17.5)	4.1 (1.5, 11.2)
Practice rate (95% CI)	0.8 (0.2, 1.4)	0.2 (0.0, 0.3)	0.0 (0.0, 0.1)	5.1 (1.8, 14.7)	10.2 (0.6, 163.6)	5.4 (0.6, 48.4)
IRR (95% CI) game vs. practice	0.8 (0.3, 2.7)	3.3 (1.5, 7.2)	5.0 (0.6, 42.8)			
Fatal events rate (95% CI)	0.8 (0.2, 1.4)	0.2 (0.1, 0.4)	0.1 (0.0, 0.1)	3.4 (1.3, 9.1)	2.6 (0.3, 22.9)	1.7 (0.5, 6.3)
Non-fatal events rate (95% CI)	1.4 (0.6, 2.3)	0.5 (0.3, 0.6)	0.1 (0.0, 0.2)	3.1 (1.5, 6.4)	12.3 (3.7, 40.2)	4.3 (1.6, 11.8)
IRR (95% CI) fatal vs. nonfatal	0.5 (0.2, 1.5)	0.5 (0.3, 1.0)	0.8 (0.2, 3.0)			
Traumatic injury events rate (95% CI)	1.3 (0.5, 2.1)	0.5 (0.3, 0.7)	0.1 (0.0, 0.2)	2.5 (1.2, 5.2)	1.7 (0.2, 14.2)	3.8 (1.5, 9.7)
All catastrophic events rate (95% CI)	2.2 (1.2, 3.3)	0.7 (0.5, 0.9)	0.2 (0.1, 0.3)	3.2 (1.8, 5.7)	8.0 (3.0, 21.4)	3.2 (1.4, 6.9)

CI: confidence intervals; IRR: incidence rate ratio.

Rhabdomyolysis cluster cases were included in all applicable categories. Rates are given per 100,000 AS.

^a^ IRRs comparing youth to other sport levels were only calculated from AY2005/06 to 2019/20.

### Athlete and event characteristics

Eighty-four percent of cases occurred among males and 35% occurred in athletes 15–17 years old (Table 1). Half of all cases occurred at the high school level, followed by 25% at the collegiate level, and 23% at the youth or amateur level. The most common time of injury was in the 3-month period of April, May and June (49%) coinciding with the typical spring lacrosse season (Table 1). The majority of incidents occurred during competition (59%), followed by practice (22%), strength/weight training session (7%), other (7%), scrimmage (3%) and conditioning session (2%). Aside from general play (28%), blocking a shot was the most common player action (19%). The most common mechanism at the time of injury included contact with a lacrosse stick or ball (41%).

### Injury characteristics

Seventy-one percent of all catastrophic events involved physical trauma. Twenty-nine percent of all catastrophic events were due to an exertional medical condition. The underlying etiology of 30 (43%) incidents was either non-traumatic SCA or commotio cordis, followed by 14 (20%) spinal cord injuries, 11 (16%) other traumatic injuries, nine (13%) severe head injuries and five (7%) incidents of severe rhabdomyolysis (Table 1). Of note, two incidents classified as other traumatic injury involved a cardiac contusion. Of the 32 total cardiac events, 17 stemmed from blunt trauma to the chest, with 15 resulting in commotio cordis and two resulting in a cardiac contusion. Commotio cordis is primarily an electrical incident in which SCA occurs following blunt trauma to the chest, while a cardiac contusion occurs when blunt trauma to the chest causes structural cardiac injury [25]. The remaining cardiac incidents were due to a variety of causes including primary electrical disease (*n* = 2), congenital heart defects (*n* = 1), hypertrophic cardiomyopathy (*n* = 2), myocarditis (*n* = 1), mitral valve prolapse (*n* = 1), unspecified cardiovascular disorders (*n* = 2) and unknown causes (*n* = 6). In three (33% of all head injury cases) of the athletes who suffered head-related injuries, pre-existing arterial abnormalities were present, which ultimately contributed to their death. All five rhabdomyolysis injuries occurred among females as a single cluster during a team-sponsored and supervised weight-lifting session at the collegiate level.

Non-traumatic SCA was the most prevalent fatal injury category, representing 48% of all fatalities (Table 1). Seven of the 15 athletes who suffered from commotio cordis died (28% of all fatalities).

### Evaluation of improvements in cardiac safety

A lagged analysis demonstrated reduced fatality rates following several high profile deaths and subsequent improvements in cardiac safety beginning in AY2005/06, with the greatest reduction occurring AY2007/08 (Supplemental Table 1). When comparing rates from AY1982/83–2006/07 to AY2007/08–2019/20, fatality rates have decreased by 95% for incidents of commotio cordis (IRR = 0.05; 95% CI: 0, 0.4), 94% for SCA (IRR = 0.06; 95% CI: 0, 0.3), and 95% for the two combined (IRR = 0.05, 95% CI: 0, 0.2) (Table 3). Similarly, overall survivorship has significantly improved over the same period, demonstrating a nearly fourfold increase in the proportion of survivors of cardiac and commotio cordis-related incidents (survivorship proportion ratio = 3.9, 95% CI: 1.3, 11.4).

**Table 3.** Male cardiac fatality incidence rates and proportion survivorship comparisons before and after initiatives to improve access to AEDs from AY1982/83–2006/07 to AY2007/08–2019/20.

	# of deaths, athlete-seasons	Before initiative incidence rate (95% CI)	# of deaths, athlete-seasons	After initiative incidence rate (95% CI)	After vs. before IRR (95% CI)
Fatality rate comparisons					
Commotio cordis deaths (*n* = 6)	5, 1,306,745	0.4 (0.1, 0.7)	1, 5,753,864	0.02 (0, 0.05)	0.05 (0.01, 0.4)
Non-traumatic sudden cardiac deaths (*n* = 10)	8, 1,306,745	0.6 (0.2, 1.0)	2, 5,753,864	0.03 (0, 0.1)	0.06 (0.01, 0.3)
Total deaths (*n* = 16)	13, 1,306,745	1.0 (0.5, 1.5)	3, 5,753,864	0.05 (0, 0.1)	0.05 (0.01, 0.2)
Incidence proportion survivorship comparisons	# of survivors, total incidents	Before initiative survivorship proportion (95% CI)	# of survivors, total incidents	After initiative survivorship proportion (95% CI)	After vs. before survivorship proportion ratio (95% CI)
Commotio cordis (*n* = 14)	2, 7	0.3, (0, 0.6)	6, 7	0.9 (0.6, 1.1)	3.0 (0.9, 10.1)
Non-traumatic sudden cardiac arrests (*n* = 13)	1, 9	0.1 (0, 0.3)	2, 4	0.5 (0, 1.0)	4.5 (0.6, 36.4)
Total incidents (*n* = 27)	3, 16	0.2 (0, 0.4)	8, 11	0.7 (0.5, 1.0)	3.9 (1.3, 11.4)

CI: confidence intervals; IRR: incidence rate ratio.

Rates and 95% confidence intervals are reported per 100,000 AS.

## Discussion

From 1 July 1982 to 30 June 2020, 69 catastrophic injuries, including 25 fatalities, occurred during organized lacrosse activities in the US. Although IRs were highest at the collegiate level, the greatest number of incidents was at the high school level. The majority of incidents were traumatic in nature, were among males, and occurred during games. Physical contact, whether with a lacrosse stick, ball or another player, was the primary mechanism. Cardiac events, both commotio cordis and non-traumatic SCA, were the most common type of catastrophic event. Catastrophic injury rates decreased over the course of the study period, though this trend was not statistically significant.

Previous studies of lacrosse injuries have investigated non-catastrophic injuries, but no study has solely examined catastrophic events. While the present study examined a different subset of injuries than previous papers, its findings corroborated several injury trends that have been observed by others, including a lower injury rate at the high school versus the college level and a higher injury rate during games at the high school level [4–6,8]. Each of these findings share a common thread that other authors have identified – rates tend to be higher when play is more physical. Male lacrosse allows more physical play with higher ball speeds compared to female lacrosse, and games are more physical compared to practice [6,8,26]. Additionally, collegiate play is generally more physical than high school play, largely due to increases in the size and strength of the athletes. Incidence rate comparisons of collegiate traumatic injuries to high school and youth traumatic injuries in this study support that more physical play at the college level is likely a contributor to the higher IR (although the latter comparison was not statistically significant). However, the highest total incident count was at the high school level. A greater number of events at this level is likely reflective of the greater number of participants. Although a recent study identified the highest injury rates among males in youth play as compared to high school or NCAA men’s lacrosse [15], the current study found youth catastrophic injury rates (both male and female) to be the lowest across all categories. These differences could be due to differences in the severity of the injuries being collected, the definitions of youth play, and the defined populations at risk (e.g. males only vs. males and females in the present study) [15]. Furthermore, Kerr et al. identified that while youth boys had the highest overall injury rates, they had the lowest injury rate of time loss injuries, indicating that perhaps youth boys experience less severe injuries than high school or NCAA men’s lacrosse, which would be consistent with the findings of the present study. Even so, more research is needed to precisely determine injury rates at the youth level.

Contact with a stick or ball was the most common mechanism of injury overall (Table 1). The second most common injury mechanism was contact with another player. This is consistent with previous studies among both males and females [6,7]. A series of rule and regulation changes, including the mandatory use of protective eyewear for female players, stricter definitions for legal checking at the high school level, the prohibition of head/face targeting at the college level, and modified specifications for lacrosse balls, have been implemented over the past two decades to address the incidence of traumatic injuries in the sport of lacrosse [12,18,22,23]. Notably, although the scope of this study did not allow for examination of differences in injury rates pre- versus post-adoption of a specific rule and regulation change, the overall reduction in IRs of catastrophic injuries in lacrosse highlights improved sports safety over the study period (Supplemental Figure 1). For instance, both eye injuries that resulted in severe orbital fractures occurred in female players prior to the introduction of mandatory protective eyewear in women’s lacrosse, and no severe eye injuries have been captured since a requirement for protective eyewear was introduced by USA Lacrosse in the 2004–2005 competitive season [12].

Importantly, this study found that the most common types of catastrophic events were cardiac in nature. This finding is consistent with research across sports identifying SCA as the most common type of catastrophic injury event with male athletes at higher risk [27–30]. Indeed, SCA is the leading cause of fatalities in young athletes during sports and exercise [27–30].

Commotio cordis, considered separately from traditional SCA due to its unique traumatic mechanism and etiology, has received strong attention in the sport of lacrosse over recent decades, in large part due to the severity and media attention devoted to these catastrophic incidents. A previous study found a higher incidence of commotio cordis events in lacrosse when compared to other sports and identified several incidents that occurred despite the athletes wearing protective chest barriers [10]. Other studies have acknowledged the low survival rate of these events and identified the need for timely medical intervention to increase the likelihood of survival [31]. Commotio cordis typically arises when a firm projectile, such as a lacrosse ball, baseball or hockey puck, strikes a specific area over the chest wall at a precise moment during the cardiac electrical cycle, producing a ventricular arrhythmia [32]. In the current study, commotio cordis cases occurred at all levels of play (five youth or amateur, seven high school and three college). Interventions that span all levels of play are needed to reduce the risk of commotio cordis. The recent specification changes for the lacrosse ball adopted by the National Operating Committee on Standards for Athletic Equipment (NOCSAE) and the current requirement for all goalkeepers (2021) and all field players (2022) to wear chest protectors represent targeted interventions to mitigate these events. Although two instances of commotio cordis have occurred since the revised ball specifications went into effect (one of which occurred outside of the study period for this paper), due to prompt emergency responses and the timely use of an automated external defibrillator (AED), both instances resulted in survival. The effectiveness of chest protectors in preventing commotio cordis has been disputed over the years [33]. Continued monitoring of these incidents and future evaluation of the most recent form of chest protectors required by USA Lacrosse is needed to determine their efficacy.

Combined, non-traumatic SCA and commotio cordis comprise nearly half of all catastrophic injuries in the sport of lacrosse over the past four decades. The injury burden these incidences place on the sport highlights the need for cardiac emergency response plans and timely access to AEDs at all levels of the sport. Studies demonstrate that survival rates in young athletes with SCA can be >80% through prompt recognition, early cardiopulmonary resuscitation, and quick use of an AED [34,35]. High profile deaths in the early 2000s largely drove efforts to improve cardiac safety and AED availability beginning in AY2005/06. Outcomes for non-traumatic SCA and commotio cordis during lacrosse have significantly improved since then [36,37]. USA Lacrosse’s partnership with industry in AY2007/08 was aimed at raising awareness for these events and providing affordable access to AEDs [24]. Following AY2007/08, there was a 95% reduction in fatalities related to SCA and commotio cordis combined among males in the current study. Although these findings cannot be attributed solely to the USA Lacrosse partnership with industry, availability and timely access to AEDs have been repeatedly shown to improve outcomes following cardiac incidents [36–41].

### Limitations

Several limitations to this study should be recognized when interpreting the results. First, there is inherent difficulty in obtaining accurate counts and event details using a passive surveillance system comprised of publicly available media reports. While there is mandated reporting at the college level from the NCAA, there is no mandated reporting at the youth, amateur or high school level. Although the authors performed additional ad hoc searches, there are likely events that occurred without any media coverage. This may have had a disparate impact in communities of lower socioeconomic status or of high minority populations, as such communities often receive misrepresentative and overall less media coverage [42–44]. Even in media reports that were obtained, information was often missing for several variables, including the athlete’s age (10 unknown), the athlete’s position (31 unknown), the athlete’s action at the time of injury (four completely unknown and 19 were classified as ‘general play’), and the emergency response at the time of injury (e.g. unknown athletic trainer availability/response for the majority of cases, AED availability and use unknown for the majority of cardiac cases).

Second, the likelihood of reporting an incident may have varied by sport level, and some catastrophic events, especially at the youth level, may have been missed. Thus, IRs may be underestimated.

Third, while not directly assessed in this paper, it should be noted that coverage by an athletic trainer varies by sport level, and access to varying levels of on-site medical care may have affected outcomes and survivability.

Fourth, there was limited participation data available for the youth and no data for amateur levels of play, resulting in the lack of rate calculations for amateur levels of play and no IR comparisons for youth to college or high school levels prior to AY2005/06. The authors categorized all participants 17 and under participating in middle school or non-school sponsored (club) leagues as ‘youth’ in order to provide a more comprehensive representation of injury rates that occurred in organized play among children and adolescents outside of the high school-sponsored setting. These varying definitions may have affected the IRs presented.

Lastly, catastrophic events are uncommon, and incident rate comparisons for certain strata were limited by small counts, resulting in wide, imprecise CIs for rates, proportions and ratio comparisons.

## Conclusions

Catastrophic injuries and exertional medical events in lacrosse are tragic events with the highest IR at the collegiate level. Males appear to suffer disproportionately from these injuries, particularly from cardiac incidents. Non-traumatic SCA and commotio cordis are the most common catastrophic events, although fatality rates have decreased substantially over the last four decades. Our results may inform future rule and regulation changes by sports governing bodies such as USA Lacrosse, the NFHS and the NCAA to further reduce catastrophic events and improve outcomes. Continued research to evaluate recent rule changes is necessary to determine their overall effectiveness and impact on sports safety.

## Supplementary Material

Supplemental Material

## Data Availability

Data are available upon reasonable request if approved by the responsible Institutional Review Board. Patient identifiable information cannot be shared.
