# Supplementary material for: Catastrophic injuries and exertional medical events in lacrosse among youth, high school and collegiate athletes: longitudinal surveillance over four decades (1982–2020)
Source: Ann Med. 2024 Feb 9;56(1):2311223. doi: 10.1080/07853890.2024.2311223 (PMC10860437; doi:10.1080/07853890.2024.2311223)

**Supplemental Table**

Supplemental Table 1: Male only all cardiac events with lags – incidence rates and survival proportions.

|  | **Fatal,**  **all 3 levels** |  |  |  |  |  |  |
| --- | --- | --- | --- | --- | --- | --- | --- |
| **ALL CARDIAC** | **n, participants** | **Rate** | **LL** | **UL** | **Ratio** | **LL** | **UL** |
| 1982/83-2004/05 | 9, 769013 | **1.17** | 0.41 | 1.93 | **1.00** |  |  |
| 2005/06-2019/20 | 7, 6291596 | **0.11** | 0.03 | 0.19 | **0.10** | **0.04** | **0.26** |
|  |  |  |  |  |  |  |  |
| 1982/83-2005/06 | 10, 1021422 | **0.98** | 0.37 | 1.59 | **1.00** |  |  |
| 2006/07-2019/20 | 6, 6039187 | **0.10** | 0.02 | 0.18 | **0.10** | **0.04** | **0.28** |
|  |  |  |  |  |  |  |  |
| 1982/83-2006/07 | 13, 1306745 | **0.99** | 0.45 | 1.54 | **1.00** |  |  |
| 2007/08-2019/20 | 3, 5753864 | **0.05** | 0 | 0.11 | **0.05** | **0.01** | **0.18** |
|  |  |  |  |  |  |  |  |
| 1982/83-2007/08 | 13, 1624753 | **0.80** | 0.37 | 1.24 | **1.00** |  |  |
| 2008/09-2019/20 | 3, 5435856 | **0.06** | 0 | 0.12 | **0.07** | **0.02** | **0.24** |
|  | **Fatal and nonfatal,**  **all 3 levels** |  |  |  |  |  |  |
| **ALL CARDIAC** | **# survivals, total**  **incidents** | **%** | **LL** | **UL** | **Ratio** | **LL** | **UL** |
| 1982/83-2004/05 | 2, 11 | 18.2% | 0% | 41.0% | **1.00** |  |  |
| 2005/06-2019/20 | 9, 16 | 56.3% | 31.9% | 80.6% | **3.09** | 0.82 | 11.65 |
|  |  |  |  |  |  |  |  |
| 1982/83-2005/06 | 2, 12 | 16.7% | 0% | 37.8% | **1.00** |  |  |
| 2006/07-2019/20 | 9, 15 | 60.0% | 35.2% | 84.8% | **3.60** | 0.95 | 13.62 |
|  |  |  |  |  |  |  |  |
| 1982/83-2006/07 | 3, 16 | 18.8% | 0% | 37.9% | **1.00** |  |  |
| 2007/08-2019/20 | 8, 11 | 72.7% | 46.4% | 99.0% | **3.88** | 1.31 | 11.45 |
|  |  |  |  |  |  |  |  |
| 1982/83-2007/08 | 5, 18 | 27.8% | 7.1% | 48.5% | **1.00** |  |  |
| 2008/09-2019/20 | 6, 9 | 66.7% | 35.9% | 97.5% | **2.40** | 1.00 | 5.77 |

**Supplemental Figure**

Supplemental Figure 1. Frequency and incidence rate of cardiac events, spinal cord injuries, and catastrophic head injuries by year that occurred in the US from July 1, 1982, through June 30, 2020.


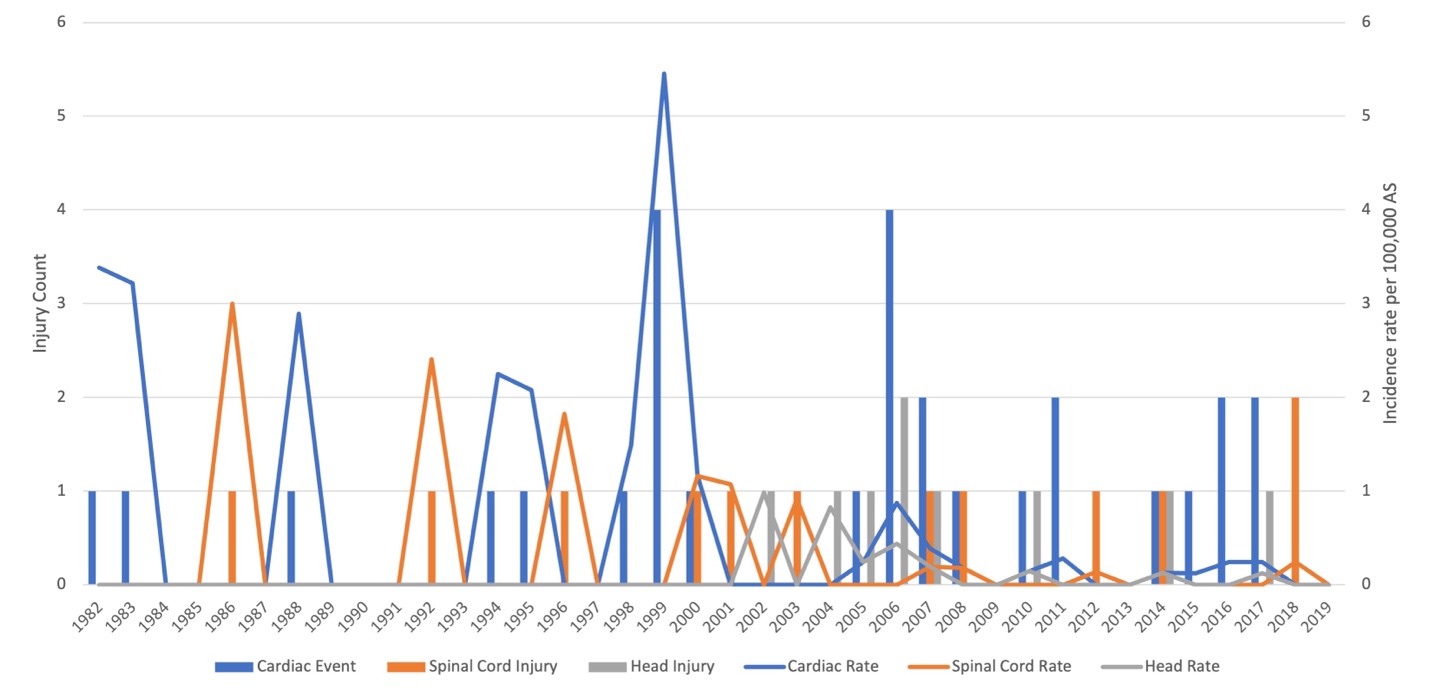

Supplement: Supplemental Material [file IANN_A_2311223_SM9060.docx]
